# Hourly level analysis of the effects of temperature extremes on emergency ambulance calls

**DOI:** 10.7189/jogh.15.04137

**Published:** 2025-05-09

**Authors:** Hao Zheng, Jian Cheng, Mingzhi Zhang, Zhen Ding, Yan Xu, Yankai Xia

**Affiliations:** 1 State Key Laboratory of Reproductive Medicine and Offspring Health, School of Public Health, Nanjing Medical University, Nanjing, China; 2 Key Laboratory of Modern Toxicology of the Ministry of Education, School of Public Health, Nanjing Medical University, Nanjing, China; 3 Department of Environmental Health, Jiangsu Provincial Centre for Disease Control and Prevention, Nanjing, China; 4 Department of Epidemiology and Biostatistics, School of Public Health, Anhui Medical University, Hefei, China; 5 Jiangsu Provincial Medical Key Laboratory of Pathogenic Microbiology in Emerging Major Infectious Diseases, Jiangsu Provincial Centre for Disease Control and Prevention, Nanjing, China

## Abstract

**Background:**

Emergency health outcomes could occur within days after exposure to temperature extremes, but knowledge about the health impact within hours after exposure is limited. We aimed to examine the transient effects of temperature extremes on emergency ambulance calls (EACs) at an hourly level.

**Methods:**

We obtained hourly data on EACs, weather conditions, and air pollutants from Nanjing, a megacity in eastern China, during 2018–21. We first extracted data from the cold and warm seasons to quantify the hourly impact of extreme cold and heat on EACs and potential modifying factors using a distributed lag nonlinear model. Then, we used a random-effects meta-analysis model to pool the estimates of extreme temperatures on EACs from this Nanjing study and from studies identified in the published literature.

**Results:**

The results showed that hourly exposure to extreme cold and heat increased the risk of EACs for all non-accidental causes, with relative risks (RRs) of 1.175 (95% confidence interval (CI) = 1.135, 1.216) at lag seven to 22 hours and 1.096 (95% CI = 1.048, 1.146) at lag zero to 10 hours, respectively. Stronger extreme heat-EAC associations were found between 16:00–24:00. The meta-analysis, which additionally included four prior studies, confirmed a significant association between hourly exposure to temperature extremes and EAC risk (RR = 1.155 for extreme cold and RR = 1.172 for extreme heat).

**Conclusions:**

Our findings indicate that transient exposure to temperature extremes can increase the demand for EACs within a few hours, which may have implications for improving ambulance service efficiency and developing an ambulance early warning system under extreme weather conditions.

Temperature-related health effects include a broad spectrum of illnesses ranging from acute to chronic symptoms. Current evidence on temperature-health early warning systems mainly derives from the findings of temperature-mortality or hospitalisation. Numerous epidemiological studies have shown that temperature extremes may increase the risk of disease mortality [1–3] or hospital-based morbidity [4–6], exhibiting a U-, V-, or J-shaped exposure-response curve in different countries or regions. However, mortality and hospitalisation may not be suitable in acute symptom assessments [7]. Emergency ambulance calls (EACs) are typically considered prehospital health events, providing real-time morbidity information [8]. Examining the temperature-EAC association is essential for understanding the acute effects of temperature on health, as it may provide earlier indicators of temperature-related health impacts [9].

Studies have suggested that acute health outcomes could occur immediately after exposure to abnormal temperatures [10,11]. Most previous studies used a daily temporal resolution to estimate the associations between temperature and EACs [12,13] or emergency ambulance dispatches [10,14], failing to fully reflect the effect of temperature variation within a day and possibly missing important information on acute health effects on a sub-daily timescale [15]. An hourly level analysis could be more suitable for elucidating the acute health effects of temperature and improving ambulance services in emergencies. Due to the issue of hourly data availability, few studies have been conducted to examine the hourly association between temperature and EACs, with inconsistent findings. For instance, a study in Luoyang, China, found that extreme cold may increase the risk of EACs for respiratory morbidity at lag 123–170 hours [16]. In contrast, another study in Taiwan, China, reported that emergency ambulance dispatches for respiratory distress peaked at zero to two hours after exposure to extreme cold [11]. Moreover, the modifying factors for hourly associations, which have important implications for optimising ambulance service, have not been fully elucidated. These previous findings prompted us to further examine the hourly magnitude of temperature on emergency ambulance outcomes and potential modifying risks.

In this study, we used the hourly EACs, weather factors, and air pollutants in an eastern Chinese city (Nanjing) to quantify the hourly effects of ambient temperature on EACs and to pool all the current epidemiological evidence to obtain an overall estimate.

## METHODS

### Study area

We conducted this study in Nanjing, a megacity in eastern China with a population of 9.4 million in 2021 (Figure S1 in the **Online Supplementary Document**). The city has a subtropical monsoon climate with four distinct seasons and generally experiences hot summers and cold winters. Nanjing has a high socioeconomic status and gross domestic product, ranked tenth among all Chinese cities in 2021 [17].

### Data collection

We obtained hourly EAC data from the Nanjing Ambulance Service Centre (NASC) between 1 January 2018 and 31 December 2021. The NASC is the provider of ambulance transport and out-of-hospital emergency services with seventy-eight ambulance stations in Nanjing during the study period. The spatial distribution of the ambulance stations covers both urban and rural areas in Nanjing (Figure S1 in the **Online Supplementary Document**). All the EACs to ambulance stations must be recorded and reported to the NASC through an online reporting system. All records of the reporting system were carefully checked to ensure accuracy and completeness. We extracted data from the online system, including ambulance call time (date and hour), the name of the ambulance station, the sex and age of the patient, and the preliminary diagnosis for the disease. To obtain an overall estimate, we extracted the hourly records of EACs for all non-accidental causes, excluding poisoning, trauma, drowning, and so on. [18]. We included all non-accidental EACs as the recorded causes were preliminary diagnoses without coding information of the International Classification of Diseases, 10th revision.

We derived hourly meteorological data (air temperature and relative humidity) from the fixed monitoring site in Nanjing during 2018–21. We collected hourly concentrations of fine particulate matter (PM) with a diameter ≤2.5 μm (PM_2.5_), ozone (O_3_), and nitrogen dioxide (NO_2_) during the same period from the Nanjing Environmental Air Monitoring Centre. To explore the effect of extreme temperature, the analysis was restricted to the warm season (May–September) for heat effects and the cold season (November, December, and January–March) for cold effects [7,18].

### Statistical analysis

We conducted a two-stage data analysis to estimate the hourly association of extreme temperatures with EACs and potential modifying factors. In the first stage, we utilised a generalised additive model in combination with a distributed lag nonlinear model (DLNM) to quantify the associations. The second stage used a meta-analysis approach to pool the current epidemiological findings on the effect of temperature on EACs to derive an overall estimate.

### Stage one: hourly association between extreme temperatures and EACs

To account for the nonlinear and delayed effects of temperature on emergency ambulance outcomes as proposed in previous studies [7,12,19], we used a quasi-Poisson DLNM to examine the hourly effect of temperature on EACs after adjusting for time trends, relative humidity, and other potential confounding factors. The DLNM can simultaneously model nonlinear exposure-response and lagged effects of temperature-related associations [20] and is widely applied in environmental epidemiology studies [1,2]. The selection of confounding factors and degrees of freedom (df) were based on generalised cross-validation (Table S1 in the **Online Supplementary Document**) and previous studies. The model is as follows:

Log (E(Yt)) = α + β1 Cb.temp + β2 NS (time, 5) + β3 NS (RH, 3) + β4 NS (hod, 5) + β5 DOW + β6 PH + β7 COVID

where E (Yt) is the estimated hourly EAC count for all causes at hour t; α is the intercept; and β1–β7 are the coefficients of the DLNM. Cb.temp is the cross-basis term for the hourly temperature, with a natural cubic spline of two df and three df for exposure and lag responses, respectively [19]. We selected 24 hours as the maximum lag hour, considering the acute effects of hourly temperature [21]. RH is relative humidity and was set by a natural cubic spline of three df [22]. We defined a natural cubic spline of five df for both time and hod to control for long-term trends and hour sequence of a day. DOW is an indicator of the day of the week, and PH represents a public holiday to control for the holiday effect. Additionally, we used a dichotomous variable (COVID) in the model to control for the possible effects of COVID-19 pandemic [23].

We further conducted stratified analyses by sex (male and female), age group (<65 years, ≥65 years), and region (urban and rural areas). The category of urban or rural areas was according to the Chinese official definition based on their administrative status, population size, and economic development [17,24]. The rural areas are highlighted in Figure S1 in the **Online Supplementary Document**. In addition, we estimated intraday variations based on habitual behaviour, dividing the day into three time periods: early day (00:00–08:00), midday (08:00–16:00), and late day (16:00–24:00). We also assessed intraseasonal variations, categorising them into early cold (1 November–15 January), late cold (16 January–31 March), early warm (1 May–15 July), and late warm (16 July–30 September) seasons [25].

The overall cumulative curves over zero to 24 hours and lag patterns of temperature extremes are presented as the main results for the temperature-EAC relationship. To inform the health intervention strategy, we chose the temperatures at which the cumulative risk of EACs began to significantly increase as the thresholds for extreme temperatures based on the results of the statistical model. This method has been widely utilised to identify temperature thresholds in assessing health effects of ambient temperature [18,26]. We did not use the minimum morbidity temperature because we could not visualise the temperature threshold in our initial analysis. The relative risk (RR) of the 95% confidence interval (CI) of EACs at the first or 99th percentile of temperature against the respective temperature threshold was calculated to show the effects of extreme cold or heat [18,27]. We used the two-sample Z test to examine potential effect modifications between two comparable groups [28]. A meta-regression was employed to perform the linear trend estimation between multiple periods within a day [29].

### Stage two: meta-analysis of current research

To obtain the overall estimate of the effect of extreme temperatures on EACs, we followed the previously described methodological framework to pool all the risk estimates using a random-effects meta-analysis method with an empirical Bayes estimator [30].

We used keywords covering hourly temperature and emergency ambulance outcomes: ‘hourly,’ ‘temperature,’ ‘emergency,’ and ‘ambulance.’ We used combinations of these keywords in the PubMed/Medline and Web of Science databases to search for original English papers from inception to 1 November 2024. We also manually checked the references of eligible studies to search for relevant studies.

We considered a study eligible if it met the following criteria: 1) original paper, 2) explored the hourly temperature-emergency ambulance association for health outcomes, and 3) reported the effects of hourly temperature extremes on emergency ambulance outcomes. We excluded a study if it met one of the following criteria: 1) it was not an original paper, such as a systematic review or conference paper, 2) the effects of hourly temperature on emergency ambulance outcomes were not reported, 3) the outcome was associated with emergency ambulance outcomes but not with diseases, and 4) the temperature and emergency ambulance outcomes were not evaluated at an hourly level.

### Sensitivity analysis

We conducted several sensitivity analyses to check the robustness of our results, including: 1) changing the maximum lag hour (36, 48, 60 hours), 2) controlling for air pollutants (O_3_, PM_2.5_, or NO_2_) in the model, 3) changing the df (two, four, five) of relative humidity, 4) changing the df (four, six, seven) of time, and 5) changing the df (four, six, seven) of hod. We conducted all analyses using *R*, version 3.4.2 (R Core Team, Vienna, Austria) with the ‘dlnm’ package to fit the DLNM and the ‘metafor’ package to perform the meta-regression. We considered a two-sided *P*-value <0.05 statistically significant.

## RESULTS

### Descriptive analysis of EACs and environmental conditions

During the study period, a total of 299 070 EACs were recorded, including 128 524 in the cold season and 122 073 in the warm season. Of the EACs in the cold season, 57.2% were male, 53.9% were people aged ≥65 years, and 60.9% were people living in urban areas. The proportions of EACs in the warm season were 55.8% for males, 49.8% for those aged ≥65 years, and 61.5% in urban areas (**Table 1**; Table S2 in the **Online Supplementary Document**).

**Table 1:** Descriptive statistics of hourly emergence ambulance calls, meteorological variables, and air pollutants in Nanjing, during 2018–21*

	Cold season		Warm season	
**Variables**	**MD (min–max)**	**x̄ (SD)**	**MD (min–max)**	**x̄ (SD)**
Emergence ambulance calls	8 (0, 49)	8.9 (4.7)	8 (0, 133)	8.3 (4.4)
Male	5 (0, 31)	5.1 (3.2)	4 (0, 71)	4.6 (2.9)
Female	3 (0, 23)	3.8 (2.5)	3 (0, 62)	3.7 (2.4)
Age in years				
*<65*	4 (0, 23)	4.1 (2.4)	4 (0, 68)	4.2 (2.5)
*≥65*	4 (0, 27)	4.8 (3.4)	4 (0, 65)	4.1 (3.0)
Urban	5 (0, 31)	5.4 (3.2)	5 (0, 70)	5.1 (3.1)
Rural	3 (0, 25)	3.5 (2.4)	3 (0, 63)	3.2 (2.2)
Temperature in °C	8.0 (–8.1, 29.2)	8.4 (5.9)	25.9 (10.3, 37.7)	25.9 (4.3)
Relative humidity (%)	74 (13, 100)	71.8 (21.9)	80 (13, 100)	76.9 (18.7)
Air pollutants in μg/m^3^				
*O_3_*	41 (3, 213)	45.2 (31.4)	76 (4, 306)	86.4 (51.7)
*PM_2.5_*	39 (3, 288)	48.6 (34.0)	22 (2, 99)	24.5 (13.3)
*NO_2_*	42 (7, 154)	46.6 (23.4)	25 (3, 112)	28.4 (15.2)

max – maximum, MD – median, min – minimum, NO_2_ – nitrogen dioxide, O_3_ – ozone, PM_2.5_ – fine particulate matter with a diameter ≤2.5 μm, SD – standard deviation, x̄ – mean

*Presented as n unless specified otherwise.

On average, there were 8.9 hourly EACs in the cold season and 8.3 EACs per hour in the warm season. The number of EACs peaked at 10:00 in the cold season and at 9:00 in the warm season. The lowest temperature occurred at 7:00 in the cold season (Figure S2, Panel A in the **Online Supplementary Document**), and the temperature peaked at 14:00 in the warm season (Figure S2, Panel B in the **Online Supplementary Document**). The hourly average temperatures were 8.4°C in the cold season and 25.9°C in the warm season. The hourly average concentrations of O_3_, PM_2.5_, and NO_2_ were 45.2 μg/m^3^, 48.6 μg/m^3^, and 46.6 μg/m^3^, respectively, in the cold season. The average of these air pollutants was 86.4 μg/m^3^ for O_3_, 24.5 μg/m^3^ for PM_2.5_, and 28.4 μg/m^3^ for NO_2_, in the warm season.

### Hourly effects of ambient temperature on EACs

**Figure 1:**
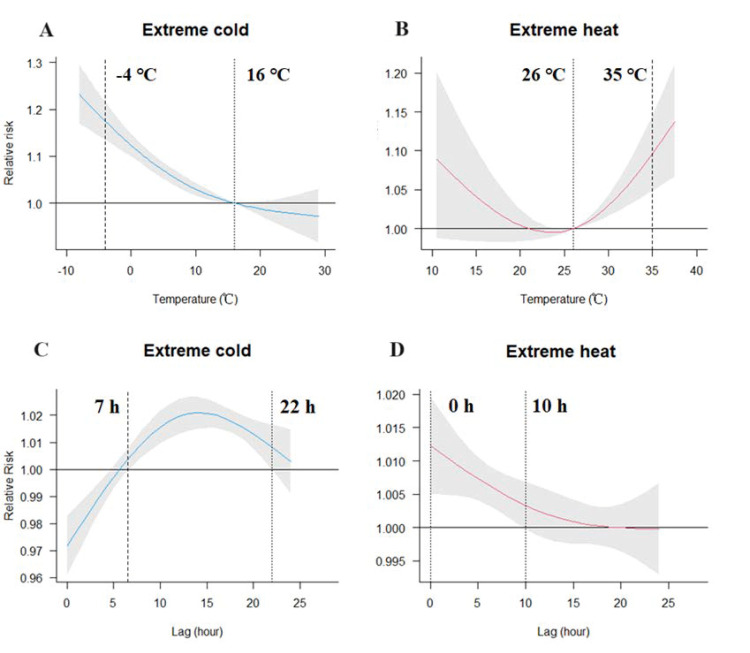
Overall hourly exposure-response curves over zero to 24 hours and lag patterns for temperature extremes and EACs for all non-accidental causes. **Panel A.** Exposure-response association for extreme cold and EACs. **Panel B.** Exposure-response association for extreme heat and EACs. **Panel C.** Lag effects of extreme cold on EACs. **Panel D.** Lag effects of extreme heat on EACs. The dashed vertical lines in the exposure-response associations represent the reference temperature and dotted vertical lines represent the extreme cold temperature (first percentile of hourly temperature) or heat temperature (99th percentile of hourly temperature). Shaded areas represent 95% confidence intervals. The lag hours between the two dotted vertical lines represent the hours that have significant adverse lag effects of extreme temperatures on EACs. EACs – emergence ambulance calls.

We observed nonlinear associations between temperature and EACs. Both cold and heat increased the risk of EACs, with thresholds of 16°C for cold (**Figure 1**, Panel A) and 26°C for heat (**Figure 1**, Panel B). We found a significant effect of extreme cold temperature (–4°C) on EACs, with an RR of 1.175 (95% CI = 1.135, 1.216) at lag seven to 22 hours and the largest RR of 1.021 (95% CI = 1.015, 1.027) at lag 14 hours (**Figure 1**, Panel C). For extreme heat (35°C), the RR was 1.096 (95% CI = 1.048, 1.146) at lag zero to 10 hours, and the highest RR was 1.012 (95% CI = 1.005, 1.020) at lag zero hours (**Figure 1**, Panel D).

The significant RRs were generally rising from lag zero to 12 hours to lag zero to 72 hours for either extreme cold or heat. For example, the RR of extreme cold was RR = 1.170 at lag zero to 12 hours, peaked at lag zero to 72 hours to RR = 1.216 (**Table 2**).

**Table 2:** Estimated effects of extreme cold (–4°C, first percentile of hourly temperature) and heat (35°C, 99th percentile of hourly temperature) on emergence ambulance calls for all non-accidental causes

Temperature (°C) and lag hour (h)	RR (95% CI)
Extreme cold	
*0–12*	1.170 (1.132, 1.209)
*0–24*	1.175 (1.135, 1.246)
*0–36*	1.185 (1.145, 1.226)
*0–48*	1.187 (1.145, 1.231)
*0–60*	1.203 (1.160, 1.248)
*0–72*	1.216 (1.170, 1.263)
Extreme heat	
*0–12*	1.077 (1.034, 1.121)
*0–24*	1.096 (1.048, 1.146)
*0–36*	1.107 (1.058, 1.158)
*0–48*	1.117 (1.065, 1.171)
*0–60*	1.123 (1.070, 1.179)
*0–72*	1.122 (1.067, 1.180)

CI – confidence interval, RR – relative risk

### Subgroup analysis

**Figure 2:**
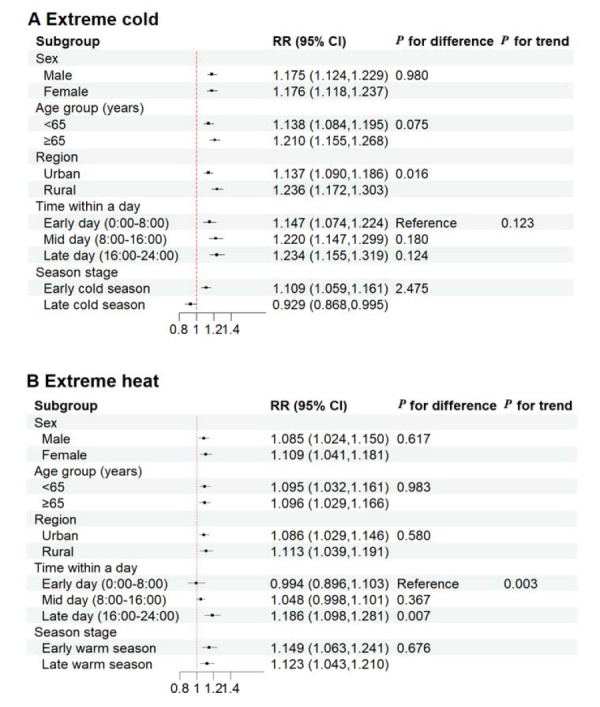
Estimated effects of extreme cold and heat on EACs for all non-accidental causes in different subgroups. **Panel A.** Extreme cold. **Panel B.** Extreme heat. EACs – emergence ambulance calls.

A significant time-dependent upward trend of the extreme heat-EAC association was found within a day (for trend *P* = 0.003). Furthermore, the effects were significantly stronger between 16:00–24:00 than between 00:00–8:00 for extreme heat (for difference *P* = 0.007). A similar upward trend of the effect of extreme cold on EACs was observed with a nonsignificant difference within a day. Exposure to extreme cold significantly increased the risk of EACs in rural areas than in urban areas (for difference *P* = 0.016) (**Figure 2**, Panel A–B). We also observed that the effect was greater in the early cold or warm season, although no significant differences were found. Additionally, significant differences by sex and age group were not observed for either cold or heat.

### Results of meta-analysis

Details of the process for the literature search are presented in Figure S3 in the **Online Supplementary Document**. Among the four additional studies included in the meta-analysis, three were conducted in China (Luoyang [16], Taiwan [11], and Chongqing [31]), and the other study was conducted in Brisbane, Australia [18]. Three studies reported the temperature-ambulance effects for both cold and heat effects [11,16,31], whereas only one study reported the heat-ambulance effect [18]. Details of the four prior studies identified in the published literature are summarised in **Table 3**.

**Table 3:** Description of the previous studies included in the meta-analysis

Author and year	Study period	Location	Temperature	Outcome	Maximum lag hour	Extreme heat, RR (95% CI)	Extreme cold, RR (95% CI)
Guo, 2017 [18]	2001–07	Australia, Brisbane	Heat	Non-accidental causes	48	1.290 (1.150, 1.450)	NA
Cui et al., 2020 [16]	2014–16	China, Luoyang	Cold and heat	Non-accidental causes	170	1.090 (1.020, 1.160)	1.210 (1.160, 1.260)
Wang et al., 2021 [11]	2006–15	China, Taiwan	Cold and heat	Respiratory distress, coma, unconsciousness, and out-of-hospital cardiac arrest	96	1.150 (1.049, 1.261)	1.104 (0.962, 1.266)
Chen et al., 2023 [31]	2019–21	China, Chongqing	Cold and heat	Injuries	72	1.210 (1.127, 1.300)	1.220 (1.063, 1.400)

CI – confidence interval, NA – not available, RR – risk ratio

**Figure 3:**
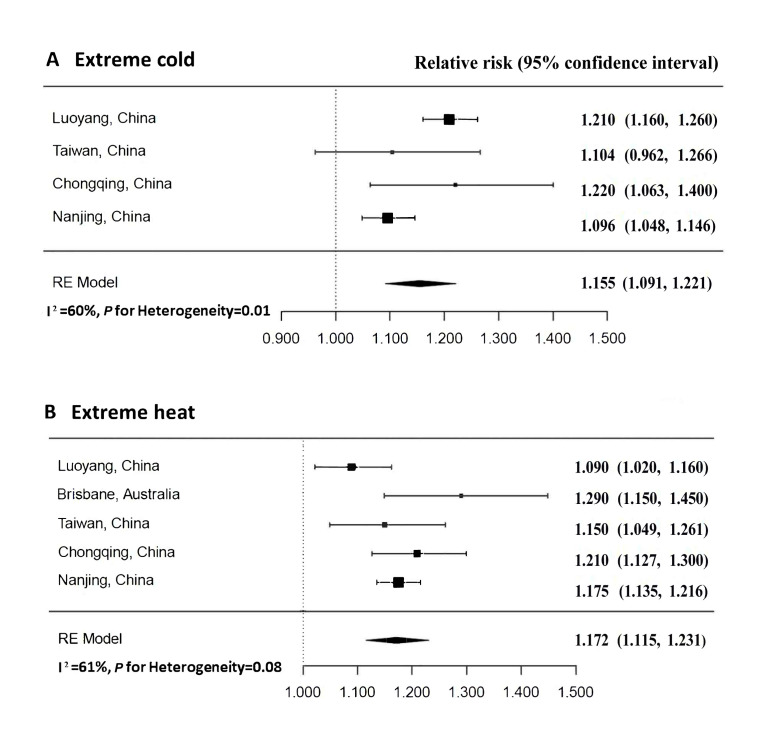
Pooled hourly effects of extreme temperatures on emergency ambulance outcomes. RR – relative risk.

The pooled associations of temperature with EACs are shown in **Figure 3**. Heterogeneity was assessed using the *I^2^* statistic. We did not observe significant heterogeneity of pooled effects for extreme heat (*I^2^* = 61; *P* = 0.08). However, significant heterogeneity in the current literature existed for extreme cold (*I^2^* = 60; *P* = 0.01). To address the observed heterogeneity, we employed a random-effects meta-analysis of the published literature included four previous studies from four other cities. The results confirmed the significant effects of extreme temperatures on EACs. The pooled RR = 1.155 (95% CI = 1.091, 1.221) for extreme cold and RR = 1.172 (95% CI = 1.115, 1.231) for extreme heat.

### Sensitivity analysis

Sensitivity analyses showed that the results were generally robust (Figure S4–5 in the **Online Supplementary Document**). The overall exposure-response curves became approximately linear when using zero to 36 hours or zero to 48 hours for extreme cold. The extreme cold effect slightly decreased after including O_3_ in the model or using a df of six or seven for time. For extreme heat, the effect slightly increased when using a df of seven for time.

## DISCUSSION

Using hourly EACs and environmental data, our study comprehensively examined the ultrashort-term effects of temperature extremes on EACs for all non-accidental causes in Nanjing, China, between 2018–21. Significant hourly associations were observed, and the temperature thresholds of detrimental effects for extreme cold and heat were identified. The adverse effects of extreme cold or heat could immediately occur within a few hours and vary within a day. Meta-analysis further indicated the hourly significant effects of extreme temperatures on EACs.

Several studies that investigated temperature-daily emergency ambulance associations have shown the considerable detrimental impacts of non-optimum temperatures in China [12,32], the UK [10], Italy [33] and Australia [8,14]. For the hourly level, of the four studies conducted in four single cities, three [16,18,31] reported significant associations between extreme cold or heat and EACs, which is consistent with our findings. Only one study performed in Taiwan, China, showed no significant impact of extreme cold on ambulance dispatches for respiratory distress [11]. This difference may be due to the different climatic conditions, disease causes, and demographic characteristics.

Identification of temperature thresholds for adverse effects and lag patterns is important to develop a temperature-related early warning system and improve the rapid ambulance response. Most studies used the minimum morbidity temperature as the threshold, which is different from the method we used. Guo et al. [18] reported that hourly heat over 27°C could immediately increase EACs in Brisbane, Australia, which is similar to the identified threshold of 26°C for extreme heat in our study. Consistent with previous studies, our study indicated that extreme heat had an immediate effect at lag zero to 10 hours. Cui et al. [16] found that extreme heat increased the risk of EACs for cardiovascular diseases at lag zero to nine hours in Luoyang, China. Additionally, a study in England and Wales [15] reported a significant association between high temperatures and myocardial infarction within several hours (zero to six hours). Xu et al. reported an elevated risk of blood pressure after exposure to the hourly temperature at lag zero to five hours [34]. Our findings demonstrated that heat exposure may trigger immediate disease onset in emergency situations. This suggests the necessity to increase the response speed of ambulance services, and more ambulance resources should be allocated during extreme hot hours.

Some plausible biological mechanisms may explain the increased risk of EACs due to abnormal temperatures. Studies have shown that exposure to extreme cold is linked to increased blood pressure, blood glucose, platelet count, and low-density lipoprotein, elevating cardiovascular disease risk [35,36]. Low temperatures can significantly suppress the human immune system by reducing lung function and vital capacity, which favours the survival of respiratory viruses [37]. Transient exposure to extreme heat may result in electrolyte imbalance and dehydration, increased heart rate, increased blood cholesterol and blood viscosity, decreased blood pressure, and increased red blood cell and platelet counts [38]. High temperatures also promote the growth of allergens (*e.g.* mould, mites, and pollen) and affect the transmission process of respiratory viruses [39].

A notable finding of our study is that there was a greater risk between 16:00–24:00 within a day, and a significant time-dependent trend was found for extreme heat. The findings of other studies have indicated that extreme heat at nighttime may lead to stronger health impacts than during the daytime [40,41]. A plausible explanation is that elevated nighttime temperature may interfere with the normal sleep regulation [41]. A reduction in core body temperature represents a critical physiological signal for the initiation of sleep onset [42]. This cooling process is driven by enhanced heat loss through the skin, which facilitates the evening decline in core body temperature [43]. However, high ambient temperatures during the night can interfere with circadian thermoregulation by impeding the efficient dissipation of core heat, thereby disrupting the natural sleep-wake cycle [44]. This disruption may increase susceptibility to chronic diseases [41,45], systemic inflammation [45,46], and psychological and cognitive impairments [47]. Since changes in emergency ambulance call risk due to temperature at different periods within a day have not been reported in previous studies, further research is required to better understand the significant association of extreme heat and EACs during this period (16:00–24:00). The urban-rural disparity in the impact of extreme cold on EACs observed in our study may be attributed to the lack of indoor heating measures in rural areas, as Nanjing is not a city with central heating during winter. Another potential explanation is the limited access to health care resources in rural areas, limiting the ability to respond effectively to extreme low temperature [48]. Similar to the evidence on mortality or hospital admissions [25], the risk of EACs was more pronounced in the early cold and warm seasons. This may be explained by the fact that people are not acclimatized to the temperature change in the early stage of winter or summer. Adaptive behaviours such as reduced outdoor activities, use of air conditioners and wearing lighter or heavier clothes may decrease the risks in the late stages of seasons [25]. The findings suggest that reasonable deployment of emergency ambulance services and protective measures should be taken to reduce the harmful impacts of extreme temperatures during high-risk periods.

In the meta-analysis, significant pooled effects of extreme temperature on EACs were observed with moderate heterogeneity. Potential sources of heterogeneity could be ascribed to the limited studies and regions included in the meta-analysis. For instance, three of four additional studies were from China. Future research should aim to include more regions and standardise statistical method to reduce heterogeneity.

Several limitations need to be noted. First, the environmental exposure data that mainly came from the fixed monitoring station may lead to exposure misclassification. Second, underreporting or missing EACs might occur when emergency systems may be overwhelmed under extreme weather events, which may underestimate the impact of extreme temperatures. Third, the main analysis was conducted in a single city, limiting our findings to other regions with different climates, urbanisation levels, or health care systems. Although our pooled study indicated significant hourly effects of temperature on EACs, further studies are required to generate more comprehensive evidence on a larger scale. Fourth, due to the issue of data limitation, other potential confounders such as socioeconomic factors, pre-existing conditions, and adaptive behaviours of patients were not considered in the study, which could influence our results. Future studies should collect as much information as possible to reduce confounding bias.

Our study also has several strengths. First, we evaluated the hourly thresholds of temperature extremes for adverse effects on EACs and found that the effects may occur immediately after exposure at the hourly level. Second, the subgroup intraday and intraseasonal analyses performed showed high-risk periods that were susceptible to adverse effects. Third, we searched all the available evidence to pool the hourly temperature-EAC associations, which contributed to reliable evidence for our results and conclusions.

## CONCLUSIONS

Transient exposures to extreme cold and heat were associated with an elevated demand for EACs at the hourly level, with a greater risk observed between 16:00–24:00 during the day for extreme heat. The adverse effects of extreme heat could occur immediately and last for up to 10 hours. Our findings have implications for the preparation and reasonable deployment of emergency ambulance services to help reduce the acute effects under extreme temperature conditions and save more lives.

## Additional material


Online Supplementary Document
