# Supplementary material for: Hourly level analysis of the effects of temperature extremes on emergency ambulance calls
Source: J Glob Health. 2025 May 9;15:04137. doi: 10.7189/jogh.15.04137 (PMC12061447; doi:10.7189/jogh.15.04137)
Supplement: Online Supplementary Document [file jogh-15-04137-s001.pdf]

Supplement to: Zheng H, Cheng J, Zhang M, Ding Z, Xu Y, Xia Y. Hourly level analysis of the effects of temperature extremes on emergency ambulance calls. J Glob Health. 2025;15:04137.

Table S1. Details of the selected *df* of confounding factors and corresponding generalized cross-validation value in the model.

Table S2. Counts and percentage of emergency ambulance calls in Nanjing, during 2018–21.

Figure S1. The study area and the emergency ambulance stations highlighted.

Figure S2. The averaged hourly counts of EACs and hourly temperature in Nanjing, 2018–21.

Figure S3. Flow diagram of literature search.

Figure S4. Sensitivity analysis for the overall temperature–EACs curve in the cold season after respectively adjusting O_3, $PM_{2.5}$, and NO_2 in the model, and changing the maximum lag hours (36, 48, 60), the *df* (2, 4, 5) of relative humidity, the *df* (4, 6, 7) of time, and the *df* (4, 6, 7) of hod in Nanjing, China.

Figure S5. Sensitivity analysis for the overall temperature–EACs curve in the warm season after respectively adjusting O_3, $PM_{2.5}$, and NO_2 in the model, and changing the maximum lag hours (36, 48, 60), the *df* (2, 4, 5) of relative humidity, the *df* (4, 6, 7) of time, and the *df* (4, 6, 7) of hod in Nanjing, China.

Table S1. Details of the selected *df* of confounding factors and corresponding generalized cross-validation value in the model

Confounding factors	Generalized cross-validation value	
	Extreme cold	Extreme heat
RH (relative humidity)		
df = 2	1.325	1.279
df = 3	1.325	1.279
df = 4	1.325	1.279
time		
df = 3	1.332	1.287
df = 4	1.332	1.281
df = 5	1.325	1.279
hod (hour sequence of a day)		
df = 3	1.665	1.542
df = 4	1.501	1.441
df = 5	1.325	1.279

Abbreviation: *df* – degrees of freedom

Table S2. Counts and percentage of emergency ambulance calls in Nanjing, during 2018–2021

Variables	Cold season		Warm season	
	Count (n)	Percentage (%)	Count (n)	Percentage (%)
Total	128,524	100.0	122,073	100.0
Male	73,508	57.2	68,077	55.8
Female	55,016	42.8	53,996	44.2
Age <65 (years)	59,245	46.1	61,302	50.2
Age ≥65 (years)	69,279	53.9	60,771	49.8
Urban	78,304	60.9	75,049	61.5
Rural	50,220	39.1	47,024	38.5

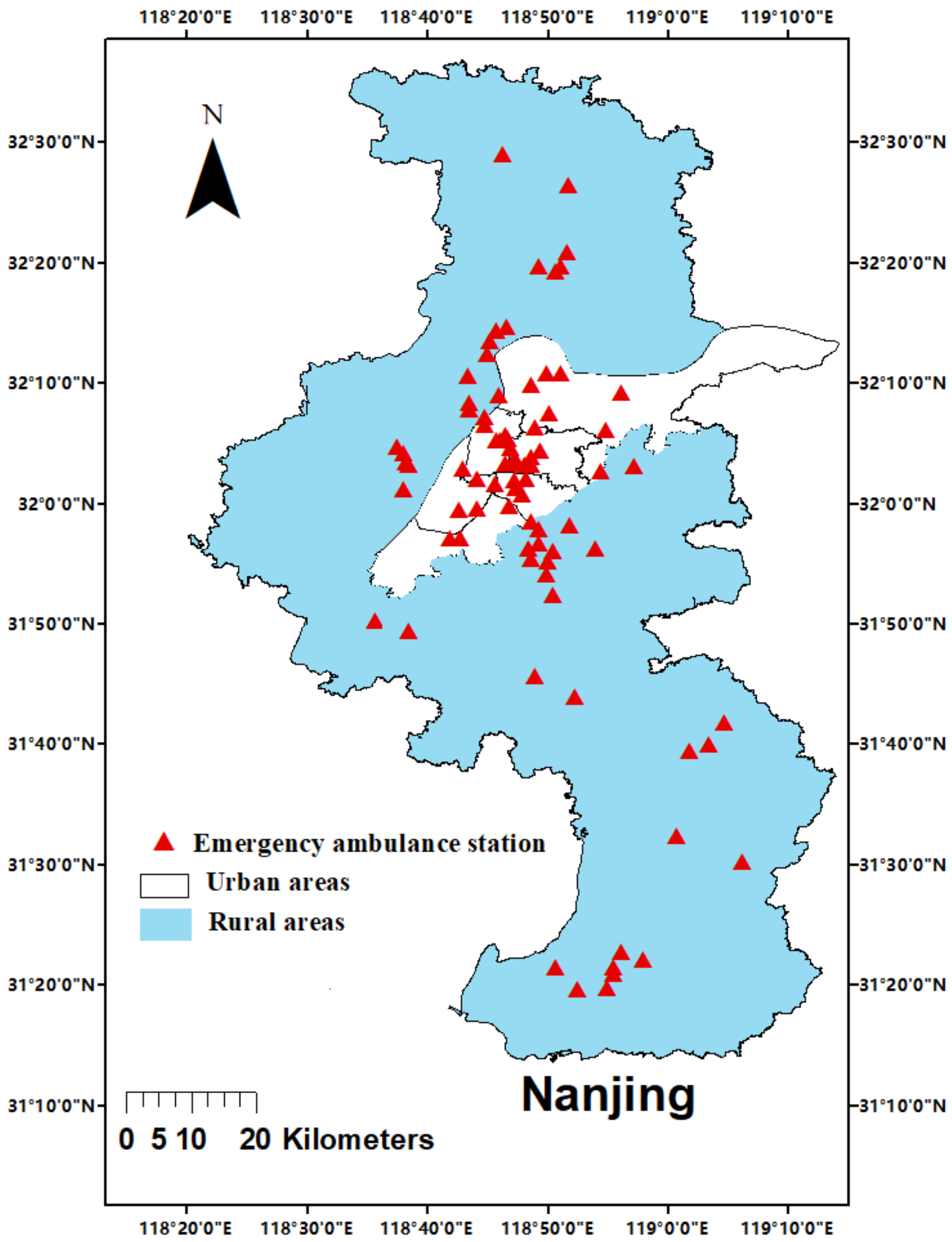

Figure S1. The study area and the emergency ambulance stations highlighted

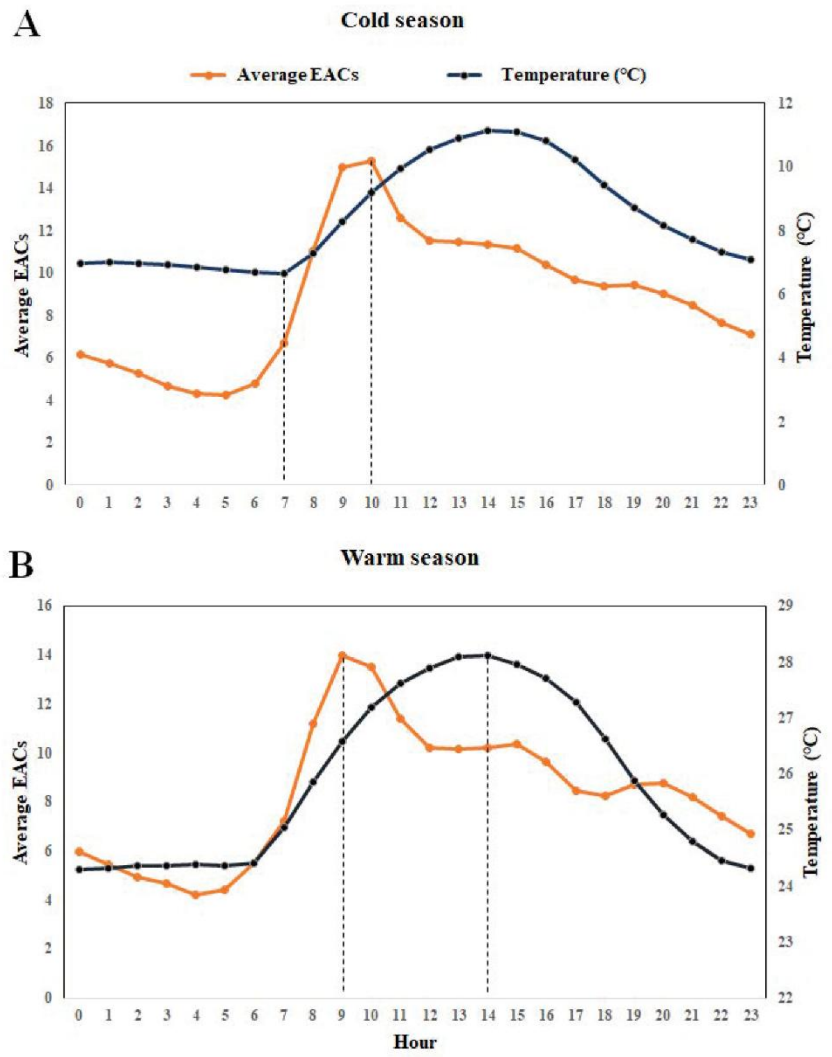

Figure S2. The averaged hourly counts of EACs and hourly temperature in Nanjing, 2018–21. **Panel A.** Cold season. **Panel B.** Warm season. The dashed vertical line indicates the highest hourly average EACs (cold and warm seasons), the highest hourly average temperature (warm season), and the lowest hourly average temperature (cold season). EACs – emergence ambulance calls.

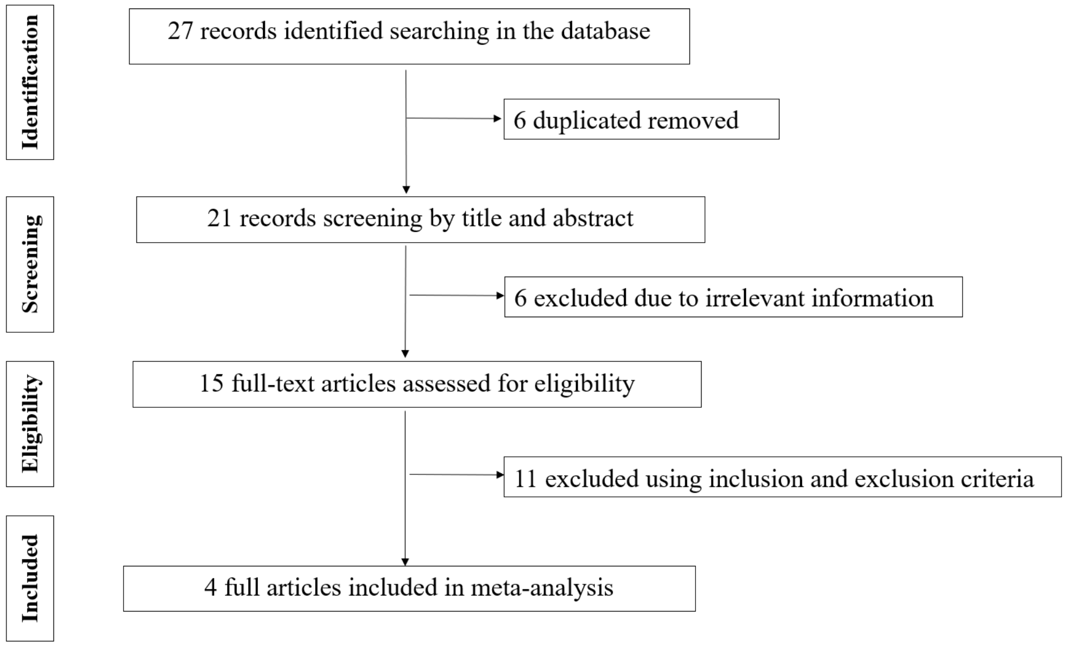

Figure S3. Flow diagram of literature search

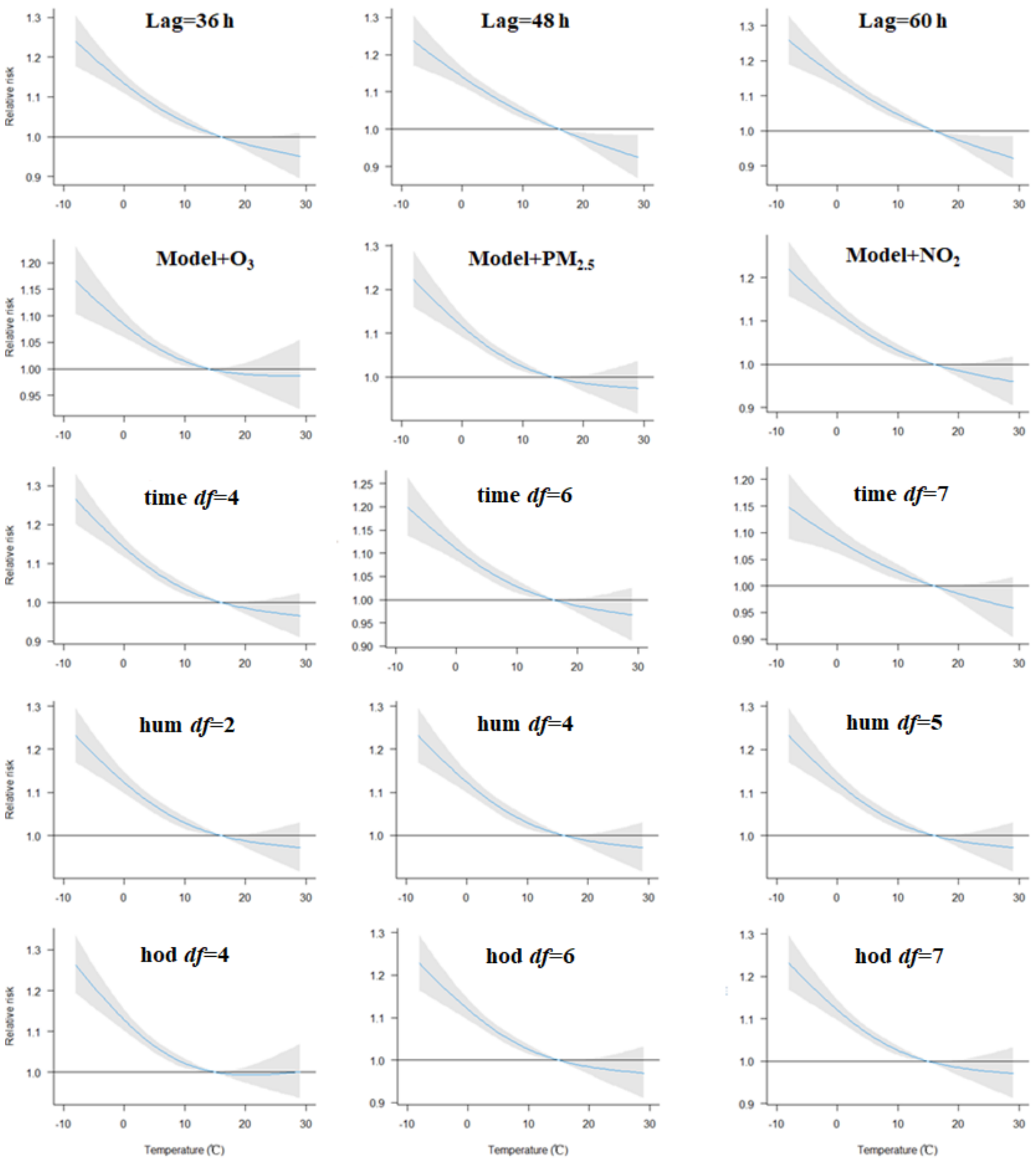

Figure S4. Sensitivity analysis for the overall temperature–EACs curve in the cold season after respectively adjusting O_3, $PM_{2.5}$, and NO_2 in the model, and changing the maximum lag hours (36, 48, 60), the *df* (2, 4, 5) of relative humidity, the *df* (4, 6, 7) of time, and the *df* (4, 6, 7) of hour in Nanjing, China.

Abbreviation: EACs – emergence ambulance calls; *df* – degrees of freedom

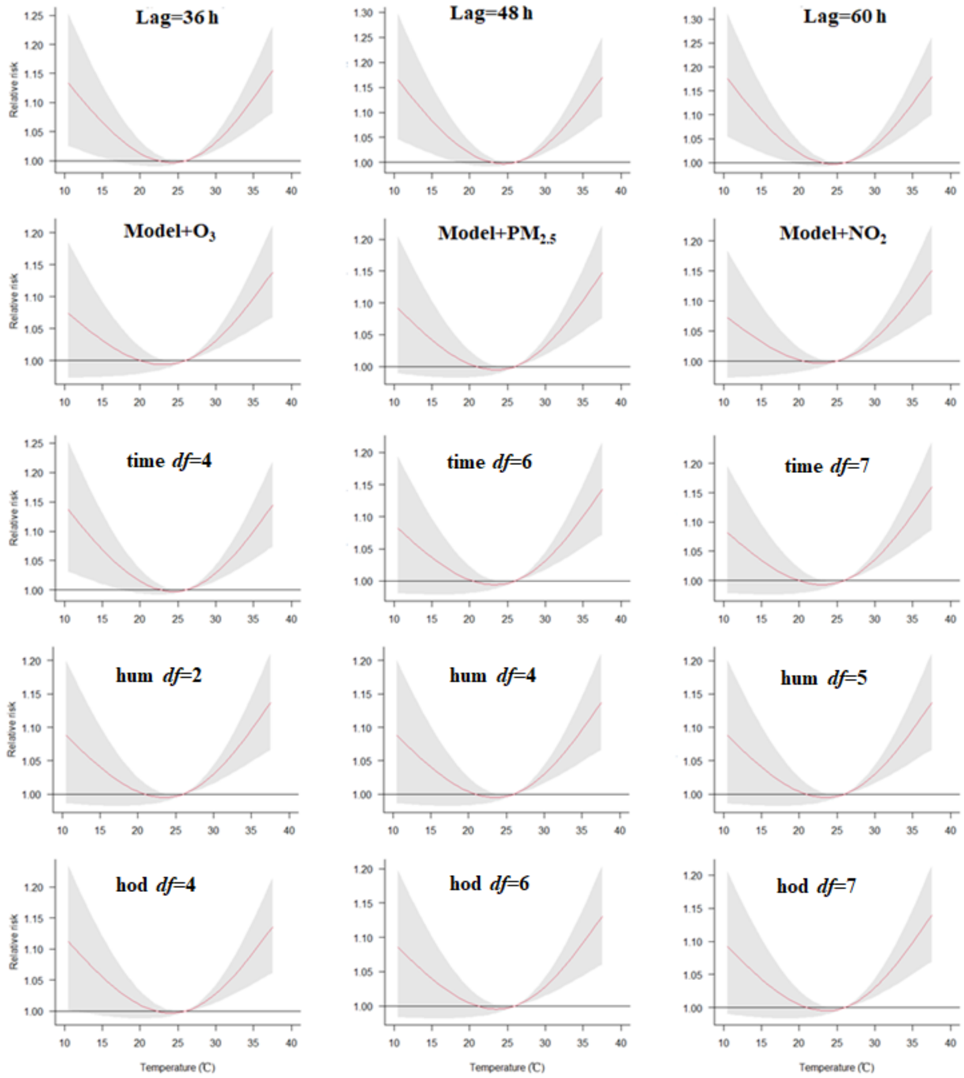

Figure S5. Sensitivity analysis for the overall temperature–EACs curve in the warm season after respectively adjusting O_3, $PM_{2.5}$, and NO_2 in the model, and changing the maximum lag hours (36, 48, 60), the *df* (2, 4, 5) of relative humidity, the *df* (4, 6, 7) of time, and the *df* (4, 6, 7) of hour in Nanjing, China.

Abbreviation: EACs – emergence ambulance calls; *df* – degrees of freedom
